# A comprehensive review of imaging findings in COVID-19 - status in early 2021

**DOI:** 10.1007/s00259-021-05375-3

**Published:** 2021-05-01

**Authors:** Ali Afshar-Oromieh, Helmut Prosch, Cornelia Schaefer-Prokop, Karl Peter Bohn, Ian Alberts, Clemens Mingels, Majda Thurnher, Paul Cumming, Kuangyu Shi, Alan Peters, Silvana Geleff, Xiaoli Lan, Feng Wang, Adrian Huber, Christoph Gräni, Johannes T. Heverhagen, Axel Rominger, Matthias Fontanellaz, Heiko Schöder, Andreas Christe, Stavroula Mougiakakou, Lukas Ebner

**Affiliations:** 1 Department of Nuclear Medicine, Inselspital, Bern University Hospital, University of Bern, Freiburgstr. 18, CH-3010 Bern, Switzerland; 2 Department of Biomedical Imaging and Image-guided Therapy, Medical University Vienna, Vienna, Austria; 3 Department of Radiology, Meander Medical Center, Amersfoort, Netherlands; 4 Department of Medical Imaging, Radboud University, Nijmegen, Netherlands; 5 School of Psychology and Counselling, Queensland University of Technology, Brisbane, Australia; 6 Department of Diagnostic, Interventional and Pediatric Radiology, Inselspital, Bern University Hospital, University of Bern, Bern, Switzerland; 7 Clinical Institute of Pathology, Medical University of Vienna, Vienna, Austria; 8 Department of Nuclear Medicine, Union Hospital, Tongji Medical College, Huazhong University of Science and Technology, Wuhan, China; 9 Department of Nuclear Medicine, Nanjing First Hospital, Nanjing Medical University, Nanjing, Jiangsu China; 10 Department of Cardiology, Inselspital, Bern University Hospital, University of Bern, Bern, Switzerland; 11 ARTORG Center for Biomedical Engineering Research, University of Bern, Bern, Switzerland; 12 Department of Emergency Medicine, Inselspital, Bern University Hospital, University of Bern, Bern, Switzerland; 13 Molecular Imaging and Therapy Service, Memorial Sloan Kettering Cancer Center, New York, NY USA

**Keywords:** COVID-19, Corona virus, SARS-CoV-2, Imaging

## Abstract

Medical imaging methods are assuming a greater role in the workup of patients with COVID-19, mainly in relation to the primary manifestation of pulmonary disease and the tissue distribution of the angiotensin-converting-enzyme 2 (ACE 2) receptor. However, the field is so new that no consensus view has emerged guiding clinical decisions to employ imaging procedures such as radiography, computer tomography (CT), positron emission tomography (PET), and magnetic resonance imaging, and in what measure the risk of exposure of staff to possible infection could be justified by the knowledge gained. The insensitivity of current RT-PCR methods for positive diagnosis is part of the rationale for resorting to imaging procedures. While CT is more sensitive than genetic testing in hospitalized patients, positive findings of ground glass opacities depend on the disease stage. There is sparse reporting on PET/CT with [^18^F]-FDG in COVID-19, but available results are congruent with the earlier literature on viral pneumonias. There is a high incidence of cerebral findings in COVID-19, and likewise evidence of gastrointestinal involvement. Artificial intelligence, notably machine learning is emerging as an effective method for diagnostic image analysis, with performance in the discriminative diagnosis of diagnosis of COVID-19 pneumonia comparable to that of human practitioners.

## Introduction

The coronavirus disease 2019 (COVID-19) pandemic has seen an unprecedented response of the scientific community; a search in the PubMed database at the time of writing this review yields nearly 100,000 scientific papers that appeared in the span of less than 1 year. For the sake of comparison, it took nearly 14 years to accrue that many citations for the search item HIV following the start of that ongoing pandemic. The unprecedented research effort sparked by the COVID-19 pandemic has yielded substantial findings in the epidemiology, immunology, comorbidity, basic physiology and therapeutics, genetics of the COVID-19 virus, and a large literature on sociological and psychological aspects. A historical comparison with the HIV epidemic is imperfect due to the intervening exponential growth of the entire biomedical literature, which has been doubling every 10–15 years, with no sign of abating. This exponential growth of the biomedical literature holds across a broad range of topics, including infectious diseases and medical imaging, but the recent growth of the COVID-19 literature seems without historical precedent. Even confining the search to the present matter of interest, i.e., the use of medical imaging in the context of COVID-19, yields more than 5000 articles published in the course of the year. This flood of information calls for a careful extraction of the most salient findings, best accomplished by the process of systematic or narrative reviews of the literature. Reviews of radiological investigations of COVID-19 have naturally emphasized thoracic imaging [1, 2], as have reviews of findings with [^18^F]-FDG/PET [3]. A few studies have taken the broader perspective of compiling imaging findings by CT, PET, and MRI [4], while addressing the multi-systemic clinical manifestations of COVID-19.

Indeed, the protean manifestations of COVID-19 pathology are evident in studies showing associations with cardiovascular [5], gastrointestinal [6], and neurological imaging results [7], with a few articles emphasizing the multisystem imaging findings [8]. Other studies report on the use of imaging for monitoring the efficacy of treatments, such as methylprednisone [9] or viral protease inhibitor [10]. In the present review, we have compiled a broad overview of PET, CT, and MR imaging results for the diagnosis and treatment monitoring of COVID-19. Some proportion of the burgeoning literature presents case studies with atypical presentations or manifestations of the infection. However, we have attempted herein to distill the main findings that are informative about the most commonly encountered symptoms and pathologies, which naturally places our main emphasis on indices of present infection in the respiratory tract. Our objective is to depict the broader utility of medical imaging for detecting COVID-19-associated pathologies involving the nervous system and sensory, musculoskeletal, and cardiovascular systems, as well as renal, gastroenterological, and dermatological involvement.

## Chest imaging

### Computed tomography of the lungs and chest radiography

#### Indication for imaging

Rapid and accurate diagnosis of COVID-19 is critically important for treatment decisions and taking appropriate isolation measures. The real-time reverse transcription polymerase chain reaction (RT-PCR) assay represents the standard of reference for detection of viral particles. However, reports from China have suggested a rather imperfect sensitivity of RT-PCR, ranging between 60 and 97% [11–13]. While RT-PCR provides near-perfect specificity with no misidentification of other coronaviruses or respiratory infectious agents, a number of factors including the quality and handling of specimens can reduce the test sensitivity in practice. Most importantly, the viral load of SARS-CoV-2 in different tissues changes dramatically across disease stages [14]. Up to four serial false-negative RT-PCR tests have been reported with delay of definite diagnosis by up to 5.1 ± 1.5 days [15]. Furthermore, radiological findings may not occur in simple temporal association with the stage of rapid viral proliferation.

Computed tomography (CT) has unsurpassed sensitivity to detect even subtle pulmonary changes due to respiratory disease. Unsurprisingly, CT has a high sensitivity for diagnosis of COVID-19. Nevertheless, CT results are also dependent on disease stage and are likely to be negative in asymptomatic patients or in up to 56% of symptomatic patients within the first 2 days of symptom onset. The rate of false-negative CT findings decreased to 9% at 3–5 days of symptomatic disease, and to only 4% at 6–12 days after first symptoms [16]. Of note, a recent literature review indicates in particular that pediatric patients with COVID-19 infection often have normal findings to CT imaging (26.5%) [17]. Thus, a normal thoracic CT cannot exclude an SARS-CoV-2 infection, especially in the early phase of disease. However, in symptomatic patients with high clinical suspicion of COVID-19 and a negative or undetermined RT-PCR test, CT can be used to diagnose pulmonary changes that are—depending on their pattern—more or less suggestive for a SARS-CoV-2 infection. In this manner, imaging might be used to triage patients rapidly before definitive RT-PCR results are available, which could be logistically very useful, especially when health care facilities are suffering a high caseload. The implementation of chest CT for this purpose has been widely discussed. Most recommendations, such as the ACR statement as well as the position paper issued by the Fleischner society, however, advocate the use of chest CT only in particular clinical scenarios and in selected patient populations [18, 19]. Chest radiography is ordinarily part of the diagnostic work-up in many patients presenting with symptoms of pneumonia, irrespective of cause. Even after several days of COVID-19 respiratory symptoms, the chest radiograph can be negative, if parenchymal changes are too subtle to be captured or difficult to differentiate from preexisting lung disease. CT is generally superior to radiography, but also the diagnostic performance of CT varies with disease stage: false negative CT reports declined from 37% in days 0–2 to 28% in days 3–5 and 19% in days 6–9 [20], somewhat mirroring the disease progression.

A number of professional societies have published recommendations on the role of imaging in the diagnosis of COVID-19, including the European Society of Radiology together with the European Society of Thoracic Imaging (ESR/ESTI), the Fleischner Society, and the British Society of Thoracic Imaging (BSTI) [21–23]. Recommendations concur that the indication for CT imaging should be a matter of the severity of respiratory symptoms of the patients, with consideration of the local prevalence of the disease and the status of health care facilities. CT imaging is not indicated in patients with mild symptoms, but an initial chest radiograph seems appropriate to diagnose the presence and extent of pulmonary opacifications in patients with moderate to severe symptoms. CT should be reserved for high-risk patients or those suspected of having complications or worsening of respiratory status, and for patients in need of expedited case management despite a present lack of definitive RT-PCR results. Typical CT findings for COVID-19 can be hard to distinguish from those with other viral infections (e.g., influenza) or non-infectious diseases (e.g., organizing pneumonia). The accuracy of image interpretation is therefore highly dependent on the local disease prevalence at the time of investigation. Combining CT findings with laboratory findings such as white blood cell count and lymphocytopenia reportedly increases diagnostic accuracy [24, 25].

In general, one critical aspect in the management of patients during the COVID-19 pandemic is to need to ensure the safety of non-infectious patients and personnel, not only in radiology departments but also throughout the hospital. Therefore, specific measures are required in order to minimize virus dissemination and contamination of imaging equipment. The American College of Radiology recently emphasized helpful measures [26].

#### Pathogenesis of COVID-19 pneumonia

**Fig. 1:**
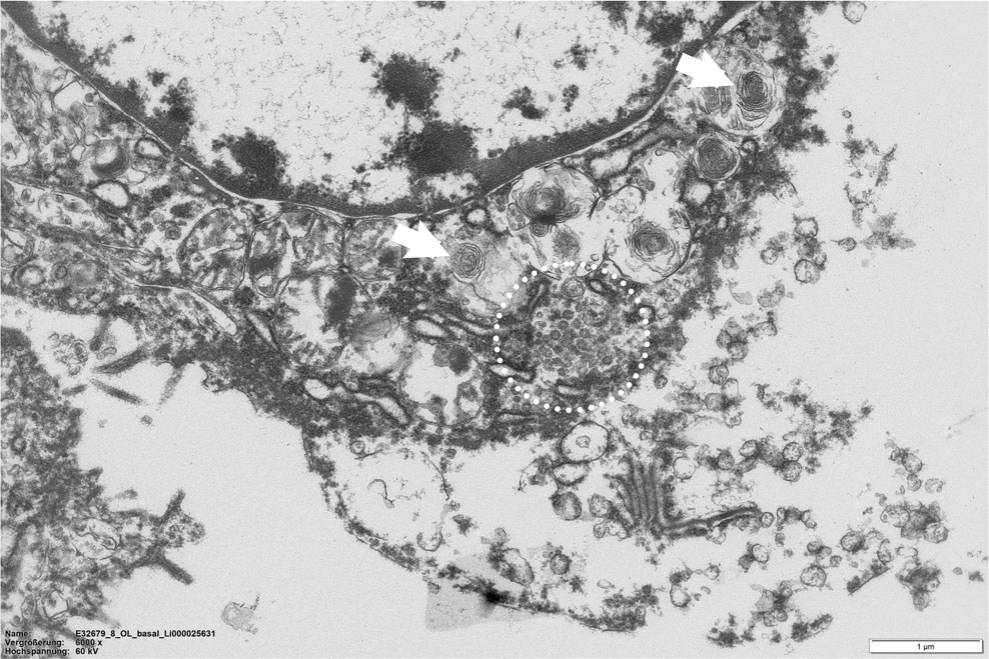
Electron transmission micrograph showing an alveolar type II cell with its characteristic lamellar bodies (arrowheads) and a group of coronaviruses (circle)

**Fig. 2:**
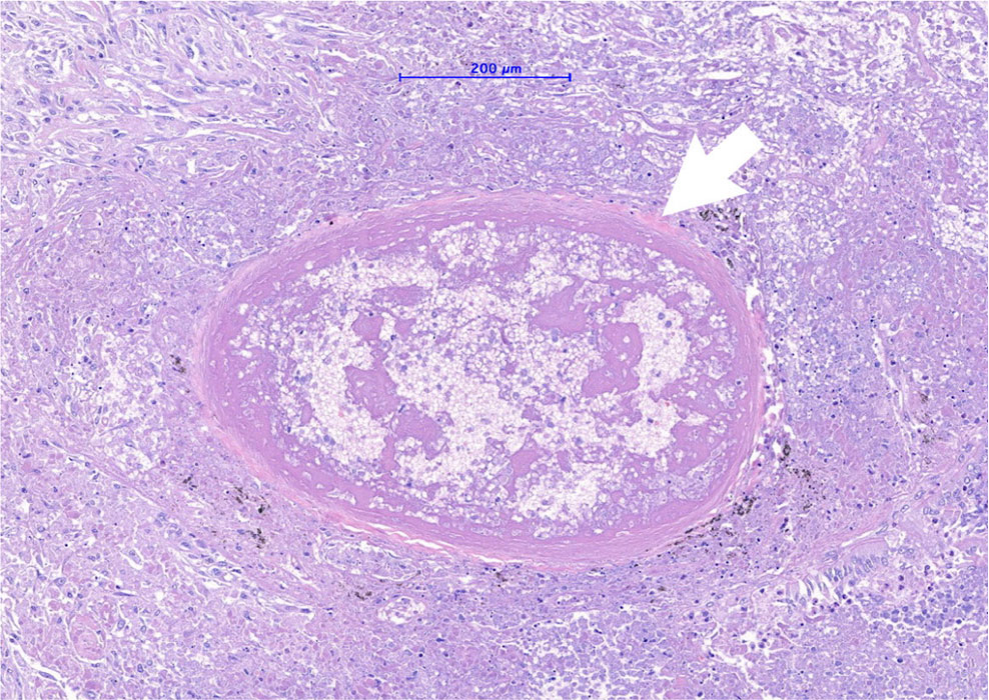
Micrograph of a hematoxylin and eosine-stained lung tissue sample of a patient with severe COVID-19 pneumonia showing a small sized artery filled with a fibrin-rich thrombus with signs of organization

Despite extraordinary efforts in the past year, the pathogenesis of COVID-19 pneumonia remains poorly understood. SARS-CoV-2 infects cells expressing the angiotensin-converting-enzyme 2 (ACE 2) receptor in their plasma membrane [27]. In humans, there is high expression of ACE 2 on ciliated epithelial cells of the respiratory tract and to a lesser degree on pneumocytes type II and alveolar macrophages, which explains the propensity of SARS-CoV-2 to cause pneumonia (Fig. 1). Autopsy and biopsy studies have demonstrated that the initial phase of COVID-19 pneumonia manifests in diffuse alveolar damage (DAD), with the formation of hyaline membranes and the desquamation of pneumocytes, as likewise observed in acute respiratory distress syndrome (ARDS) [27]. Features of acute bronchitis and bronchiolitis are also common. Furthermore, vascular damage is often seen in COVID-19 patients, presenting either as thromboembolism of larger blood vessels and/or as intravascular clot formation in capillaries that resembles neutrophilic capillaritis [27] (Fig. 2).

#### Imaging findings on chest radiographs

Chest radiographs, although not as sensitive as CT, are the diagnostic mainstay in many centers. The most commonly observed COVID-19 findings on chest radiographs are air space opacities including consolidation and ground glass opacities, also reticular abnormalities occur. These patterns have a bilateral distribution in the mid-lung field or basally with a peripheral predominance [20, 28, 29].

#### Imaging findings on chest CT

**Fig. 3:**
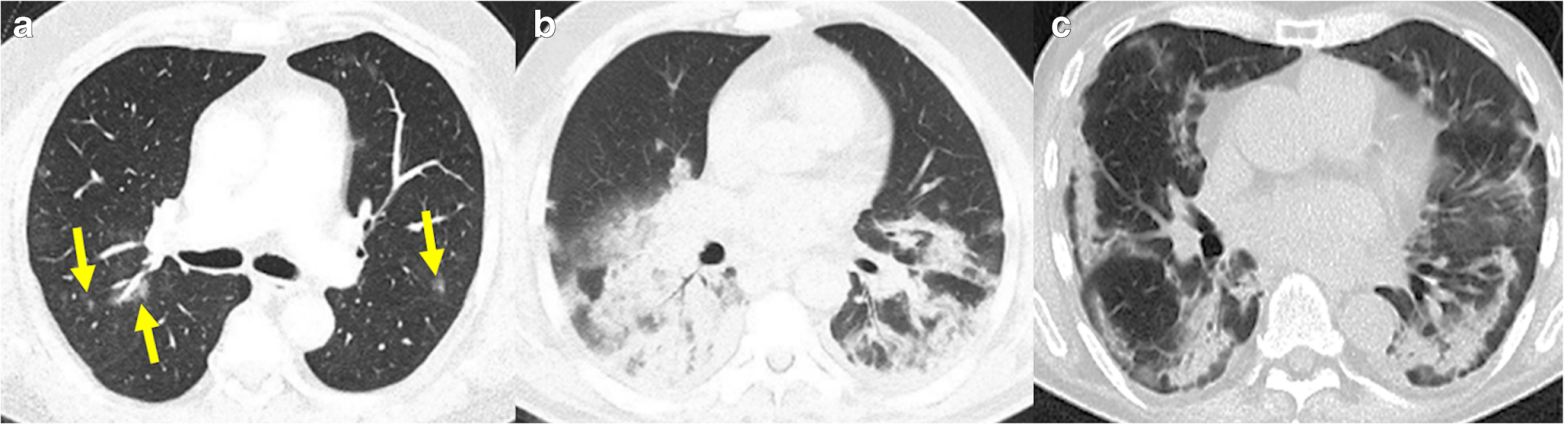
Three different patients with COVID-19 related abnormalities in the lungs shown by non-contrast CT. **a** A 55-year-old male patient with COVID-19 diagnosed 1 day before. CT showing ill-defined ground glass nodules in both lungs (yellow arrows). **b** A 49-year-old male patient with acute dyspnea for 4 days. CT showing extensive ill-defined consolidations with positive air-bronchograms in both lower lobes: COVID-19 was confirmed by RT-PCR. **c** A 60-year-old male patient with consolidations in the lung periphery sparing the subpleural space. The findings are compatible with organizing pneumonia indicating ongoing repair of the known COVID-19 pneumonia

The most common CT pattern is either isolated ground-glass opacities, which are observed in one half of COVID-19 patients, or a combination of ground-glass opacities and consolidations, which is observed in around 44% of patients (Fig. 3) [30]. These typical findings most likely reflect pulmonary edema with hyaline membrane formation, as was reported in an autopsy series [27, 31]. Isolated consolidations are observed in 24% of patients [30], with an increasing prevalence as the disease progresses. In addition to ground glass and consolidations, other typical findings in COVID-19 are engorged pulmonary vessels (64%), septal thickening (60%), and pleural thickening (42%), as well as the so-called crazy paving pattern represented by ground glass opacities in combination with underlying interlobular septal thickening, and the reversed halo sign [30, 32]. The reported distribution of the pulmonary CT findings is bilateral (79%), multifocal (70%), or sometimes patchy, with a predominance of the lung periphery and the bases of the lung [33–37]. In other patients, the abnormalities show a preferential bronchovascular (12%) or diffuse distribution (44–59%) [37–39]. Based on chest CT patterns, a grading into low, moderate or high level of suspicion for COVID-19 pneumonia has been proposed, aiming to facilitate communication of the findings [40].

Although imaging findings of pulmonary involvement are relatively characteristic of COVID-19, they are not specific for this disease. Underlying conditions might influence the imaging presentation, requiring careful consideration of any previous patient history [41–43]. Other entities such as influenza virus or respiratory syncytial virus may mimic radiological aspects of COVID-19 pneumonia [44]. The pattern of distribution of finding in the lungs as well as the composition of parenchymal opacities may assist in the discrimination between viral etiologies. For instance, influenza pneumonia is reported to present with dense opacifications, whereas COVID-19 is characterized by more prevalent ground glass density [41].

**Fig. 4:**
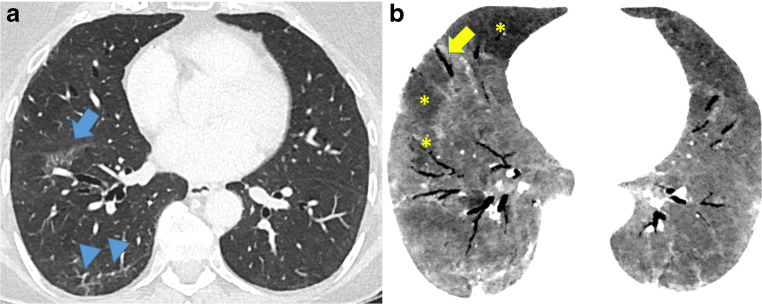
Axial CT section at the level of the lower lobes in a patient 4 months after severe COVID-19 pneumonia. Focal, subpleural reticulations with associated volume loss (image **a**, arrow) and subpleural bands (arrowheads in **a**) represent residual fibrous foci. Axial minimal intensity projections (10-mm slice thickness; image **b**) picture peripheral traction bronchiectasis. In addition, a common finding in post-COVID-19 patients is multifocal air-trapping (asterisk in **b**) pointing towards involvement of small airways

Over the course of the COVID-19 illness, the relative proportion of ground glass opacities decreases, whereas the proportion of consolidations and reticular abnormalities tends to increase [45, 46]. The extent of consolidations peaks between day 9 and 13 after the initial onset of symptoms [46, 47]. Importantly, the gradual resolution of abnormalities can take several weeks [47]. In some patients, however, the diffuse alveolar damage in COVID-19 may lead to an adult respiratory distress syndrome (ARDS) and life-threatening multi-organ dysfunction [48]. CT findings in patients with ARDS secondary to COVID-19 show extensive bilateral ground glass opacities with or without admixed consolidations [49]. An increasing number of imaging and autopsy studies also report lung fibrosis as a long-term consequence of COVID-19 with traction-bronchiectasis and fibrotic bands [50–52]. Importantly, pleural effusion, cavities, lymphadenopathy, mucous plugging, and tree-in-bud are uncommon findings in COVID-19, and their presence should thus raise the suspicion of a bacterial superinfection or other complications [53, 54]. Initial reports also address the issue of late effects in recovered patients following COVID-19 [55, 56]. Figure 4 shows lung-imaging sequelae of severe COVID-19.

#### Imaging findings on lung ultrasound

Lung ultrasound (LUS) findings in COVID-19 pneumonia depend primarily on the phase of the disease, as well as disease extent. In earlier stages, in which CT shows primarily ground-glass opacities, LUS is characterized by an increased number of the so-called B-lines, which are defined as hyperechoic lines radiating vertically from the pleura into the lung parenchyma [57–62]. The pleura frequently appears rugged [61]. As with CT, these findings are predominantly evidence in the lower lobes. With increasing severity of COVID-19 pneumonia, the number of these B-lines increases, and they may coalesce and involve larger areas of the lung. Furthermore, hypoechoic lung consolidations with or without air bronchograms may be observed [62]. Importantly, LUS findings—as also other imaging modalities—have a certain sensitivity of COVID-19, but are not specific.

LUS is used as a decision-making and disease-monitoring tool in COVID-19 patients [62]. As such, LUS serves primarily as a triage tool in the emergency room, to guide decisions about mechanical ventilation (PEEP titration) in the intensive care unit and as a monitoring tool to diagnose complications such as pleural effusions or intrapulmonary abscesses [58].

#### Prognostication of patient outcome

The aim of many prognostic studies has been to assess prospectively the risk of critical illness calling for mechanical ventilation and potentially leading to death, where the restricted availability of ventilators and staff at critical care facilities has been an important vexation. This kind of risk prediction remains quite challenging and must accommodate various factors such as comorbidities and laboratory findings, as well as treatment, which has been evolving over time in the pandemic. Thus, interpretations of the results of prognostic studies require some caution in light of these considerations.

A meta-analysis including 13 studies and 3027 patients identified age > 65, current smoking, and various comorbidities such as hypertension, diabetes, cardiovascular disease, and chronic lung disease as risk factors for progression to critical and mortal outcomes [63]. Multiple publications have described various associations between laboratory findings with poor prognosis or critical illness. Notably, elevation of CRP, LDH, Ferritin, Procalcitonin, D-dimer, Il-6, and cardiac enzymes is a characteristic of more severe cases [64, 65].

In addition to consideration of patient characteristics and clinical parameters, imaging has also played an important role in the prediction of patient outcome. Among the findings that have been proposed as indicators of disease severity are visual assessment of >25% involved lung parenchyma [66], which mirrors the threshold of less than 73% well–aerated lung parenchyma on admission chest CT [67]. In addition, several studies emerging from China have confirmed the relationship between extent of parenchymal changes on CT and patient outcome. However, these studies generally failed to consider the influence of confounding factors.

A number of CT severity scores have been proposed based on visual assessment of involved lung parenchyma, using either small increments (< 10%, 10–25%, 25–50%, 50–75%, > 75%, [21]) or assessing the involvement (0, < 50% or > 50%) per lung segment [68]. A threshold of 19.5 of 20, corresponding to about 50% of involved lung tissue, yielded a ROC area under the curve (AUC) of 0.892 for identifying severe COVID-19 disease. Artificial intelligence methods are now finding application for quantification of involved lung parenchyma, with high agreement scores with observer results [69], as we shall described in detail below.

As for CT, there are a number of publications using a reading of the admission chest radiograph (CXR) together with clinical and laboratory finings for risk predication. While Toussie et al. targeted only patients <50 years and did not consider laboratory findings [70], Liang et al. developed a risk score consisting of CXR findings and laboratory findings in a large Chinese data cohort across a broad age range. In their patients with generally less severe illness, the risk score had a very high discriminatory value (AUC 0.88) [71]. Schalekamp and coauthors developed a risk score for a more challenging group of patients with moderate to severe symptoms who were hospitalized [72]. The final risk model (*DUTCH COVID-19 risk model) included gender, COPD, symptom duration, inflammatory laboratory parameters (neutrophil count, C-reactive protein, LDH), and CXR findings. Risk-determining CXR findings were the distribution of opacifications (central/diffuse versus peripheral only) and their extent (0, < 50%, and > 50%) separately for the four quadrants of the posteroanterior radiograph. Another Dutch group has followed a similar approach of combining CXR and laboratory findings [73].

We note that the risk and occurrence of pulmonary embolism was not included in the risk models mentioned above. While concentration and increases of D-dimer have emerged as an important risk factor, we are not aware of any publications yet showing an association between the extent of parenchymal findings and the occurrence of pulmonary embolism.

### PET/CT

**Fig. 5:**
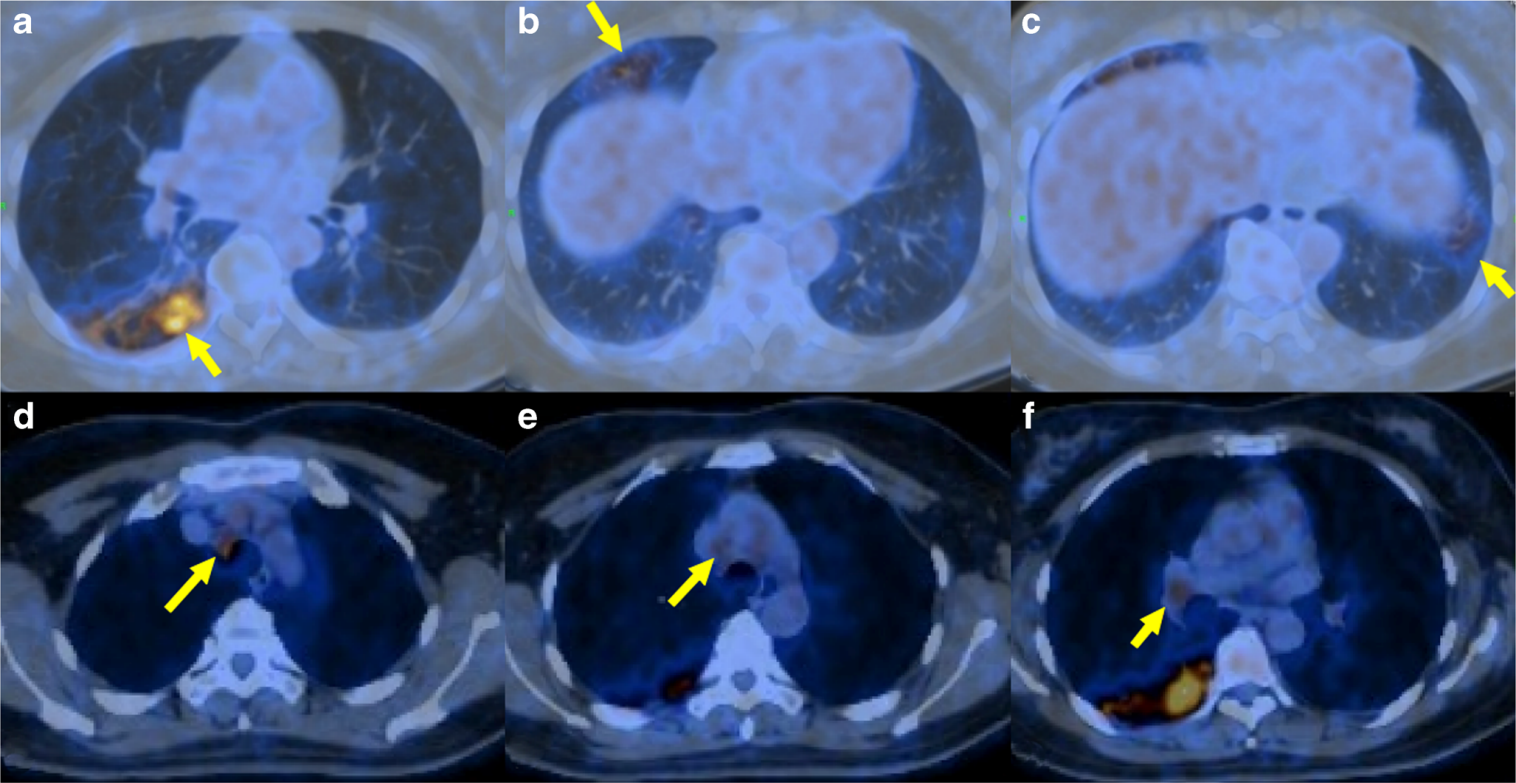
A 48-year-old female was hospitalized with fever, cough, and fatigue with a high index of clinical suspicion for COVID-19. [^18^F]-FDG PET/CT revealed high levels of uptake in the lower lobe of the right lung (**a**), the middle lobe (**b**), and in the left lower lobe (**c**) with patchy opacities. High uptake was also observed in mediastinal lymph-node stations 2R (**d**), 4R (**e**), and in the right hilum (**f**), as shown by the yellow arrows

Since the beginning of the pandemic, a number of case reports, letters to editors, and case series have been published reporting [^18^F]-FDG-PET/CT imaging findings in patients with RT-PCR-confirmed SARS-CoV-2 infection (Fig. 5). Intriguingly, as with CT, there are several case reports of PET/CT findings in otherwise asymptomatic individuals, and indications that CT can reveal sub-clinical infection. In consideration of known issues regarding the low sensitivity for RT-PCR [74–76], a number of authors have proposed CT as a potential screening tool, although official guidance remains firmly against using CT as an initial screening test. Despite the numerous case reports enthusiastically suggesting a potential role for [^18^F]-FDG-PET/CT [77, 78], there is not yet any clear evidence-based rationale for molecular imaging in the diagnosis or management of COVID-19.

A large body of historical literature exists on the molecular imaging of infection, including the use of ^67^Ga-citrate scans for AIDS-associated *Pneumocystitis jirovecii* infection. However, there have been relatively few publications on the nuclear imaging of coronavirus-associated SARS, and very few of the surfeit of recent PET/CT case reports on COVID-19 placed their findings into this wider context of respiratory infections. Indeed, a PubMed search for the terms “PET/CT” and “viral pneumonia” reveals only nine publications prior to 2019 (only two of which were truly reports of viral pneumonias). In contrast, the same search for the year 2020 yielded 72 hits. This is noteworthy, since at least three pandemics of respiratory pathogens have occurred subsequent to the advent of multimodal PET/CT imaging (SARS-CoV-1 2002–2006, H1N1pdm09 “Swine Flu” 2009–2010, and MERS-CoV 2012), not to mention the annual influenza season, which frequently leads to excess winter mortality and places significant strain upon health systems. While there are significant differences between COVID-19 and previous outbreaks, not least in its magnitude, this dearth of data prior to the current pandemic may nevertheless suggest that there had hitherto been limited clinical requirement for additional [^18^F]-FDG-PET/CT imaging in the management of individuals with viral pneumonias. Alternately, this phenomenon may indicate what has been termed a “covidisation” of research in 2020 [79]. Indeed, the preponderance of available studies is limited to case series or non-controlled observational data, and few studies have confirmed PET findings by laboratory testing or have included negative or positive controls. There is therefore a lack of data regarding the true sensitivity and specificity of [^18^F]-FDG-PET/CT for COVID-19, which calls for cautious interpretation of any suggestive findings. It is in this context that we provide a critical overview of the currently available data.

#### PET findings in viral pneumonia

The SARS-CoV-1 pandemic (2002–2004) was associated with a considerably higher individual morbidity and mortality in the acute phase than has been observed in 2020 with SARS-CoV-2. For this earlier outbreak, occurring only shortly after the advent of dual modality PET/CT in clinical routine, only sparse data using conventional imaging modalities are available. The few reports describe similar changes to those now seen SARS-CoV-2, being indistinguishable from findings in other viral pneumonias [80, 81]. There is a single case study with [^18^F]-FDG PET/CT for the 2012–2015 Middle East respiratory syndrome (MERS-CoV) pandemic. Similar to findings with SARS-CoV-2, there was increased metabolic activity associated with the ground-glass opacities in the lung [82]. The spatio-temporal pattern of [^18^F]-FDG uptake in ferrets with H1N1pdm09 virus reported by Jonsson et al. showed a correlation between lesion standardized uptake value (SUV) and bronchiolitis-related pathologic scoring, thus suggesting a relationship between viral pathophysiology and PET findings, albeit in an animal model [83]. In contrast, when assessing the suitability of [^18^F]-FDG PET/CT for the characterization of HIV-related bronchiectasis in children, Masekela et al. found no correlation between PET findings and disease activity [84]. [^18^F]-FDG PET/CT has been investigated previously as a means of therapy monitoring in respiratory infections and as a diagnostic tool in cases of cryptogenic infection, such as nosocomial or ventilator-associated pneumonia, although these data are of a preliminary nature [85]. Therefore, there is not yet any firm basis for interpreting the flurry of COVID-19-related [^18^F]-FDG PET/CT data.

#### Epidemiology of PET findings

A variety of experiences with [^18^F]-FDG PET/CT from around the world have been published, ranging from various coincidental COVID-19-related findings [86], to rare cases of positive results (23/1079 [87]), to significant findings in clinics at pandemic hotspots. The frequency of findings, when analyzed statistically and compared to epidemiological data, may provide insights into the underlying dynamics of the pandemic [86, 88]. In Brescia, Italy, a case series of 65 [^18^F]-FDG PET scans over an 8-day period in March 2020 showed six scans with findings suggestive for COVID-19 (9.2%), although, in accordance with the local guidelines, confirmatory laboratory testing was not done in all patients [89]. That same study reported a low incidence of findings suspicious of viral pneumonia in a similar 8-day period in March 2019 (*n* = 2/80, 2.5%). Likewise, in a 3-day period in March 2020 in Bergamo, Italy, 5/13 suspicious scans were identified, although again, interpretation of these findings is confounded by the lack of laboratory testing for all individuals and by the blind antibiotic therapy then initiated for presumed bacterial pneumonia in accordance with local guidelines [90]. Similar results were reported for southern Italy by Maurea et al., observing significantly more findings suspicious for COVID-19 between Feb and April 2020 compared to 2019 [91]. Maurea et al. reported their data using a standardized reporting system (CO-RADS) in the interpretation of their scans [92], whereas Pallardy et al. reported data from Nantes, France, using a French classification system, which showed a small increase in incidental findings (2.2% 2019 vs. 3.8% 2020) [93]. Notably, the presented cases included areas of minimal lung involvement and were thus of questionable clinical impact. It is unknown if such patients were in fact COVID-19 positive and, if so, whether they were still infectious at the time of scanning. In contrast to these data, Halsey et al. reported a series of scans (*n* = 160) conducted in 2020 at an academic center in London, UK; these scans were analyzed for incidental findings relative to a date-matched control group (*n* = 205) from 2019. Although a high number (16.3%) of their 2020 patients had incidental findings, there was no significant difference in frequency compared to the 2019 control group. This does suggest that such a high rate of incidental findings can be common even in “normal times,” although the merits of choosing 2019 scans as a control-group are perhaps questionable; early indications suggested that the 2019 influenza season was then already (prior to COVID-19) on track to be one of the worst in recent decades (https://www.cdc.gov/flu/about/burden/preliminary-in-season-estimates.htm.). The winter of 2019 was therefore not a “normal” baseline for comparison. In common to all these studies was the lack of laboratory testing to confirm or refute COVID-19 diagnosis, and the exclusion of symptomatic individuals. Examination protocols (free-breathing vs. deep inspiratory breath hold acquisition and reconstruction parameters) were not standardized, and are thus likely to have had a considerable influence on the results. Interpretation criteria varied between studies or relied on the readers’ own interpretation of what constitutes a suspicious finding. Levels of sub-speciality experience in the reporting of thoracic imaging may also differ between centers. There is considerable overlap between COVID-19-related radiological findings and other causes of acute lung injury or organizing pneumonia. Such suspicious findings, which these studies report to be consistent with COVID-19, may be better described under the more inclusive differential diagnosis of “viral pneumonia,” which is in-keeping with the Radiological Society of North America consensus statement [94]. Reports regarding the frequency of non-specific PET/CT findings should therefore be interpreted with a high degree of caution.

#### Pathophysiological insights

Molecular imaging may provide insights into the underlying pathophysiological characteristics of COVID-19, which, at the time of writing (12 months into the pandemic), remain poorly understood. Nassodi et al. call for the nuclear medicine community to “look beyond the obvious,” referring to incidental findings in [^18^F]-FDG PET [95]. Two case reports are available for COVID-19-related findings in [^68^Ga]-PSMA-11 PET/CT (with non PSMA-avid classical ground class opacities in the CT) [96] and in [^18^F]-fluorocholine PET/CT for recurrent prostate cancer (with fluorocholine-avid disease and lymphadenopathy present) [97]. We find no other reports of incidental COVID-19 findings with other PET tracers. [^18^F]-FDG-PET/CT is a well-established modality for the imaging of inflammation [98]. Although [^18^F]-FDG-PET/CT reports of Kawasaki-like vasculitis have been prominent in the literature [99], no case reports are available of children with COVID-19 undergoing nuclear medicine imaging with [^18^F]-FDG PET. Coagulopathy is a notable extrapulmonary feature in patients suffering from severe COVID-19 [100]. Although the molecular imaging of the coagulation system is at a preliminary stage, novel and promising techniques such as the radiolabelled derivative of factor VII, ^18^F-FVIIai [101], hold some promise as tools for future research.

### Lung scintigraphy

Dysfunction of multiple organ systems has recently been described in patients suffering from COVID-19 pneumonia [102, 103]. Various publications report coagulopathy as an accompanying manifestation of infection with SARS-CoV-2 [104]. The administration of low-molecular-weight heparin during the hospitalization of COVID-19 patients was associated with reduced mortality [105], suggesting an underappreciated impact of coagulopathy. Therefore, not only CT-angiography [106] but also ventilation/perfusion single photon emission tomography (V/Q-SPECT) may play important roles in excluding pulmonary embolism in cases of COVID-19 pneumonia, especially when there is contraindication for contrast-enhanced CT, e.g., renal failure [107–109]. According to the algorithm of Zuckier et al., lung scintigraphy should be performed only with perfusion component (*Q*) to minimize the risk of COVID-19 transmission and thus protect nuclear medicine employees [109]. On the other hand, other publications recommend performing V/Q-SPECT in COVID-19 patients to reduce the rate of false positive results, which they consider less likely to occur with V/Q-SPECT than in Q-SPECT with low-dose CT [110, 111]. The mentioned publications emphasize the importance that staff should use protective equipment such as N-95 masks during the ventilation procedure of COVID-19 patients undergoing diagnostic examination [109].

**Fig. 6:**
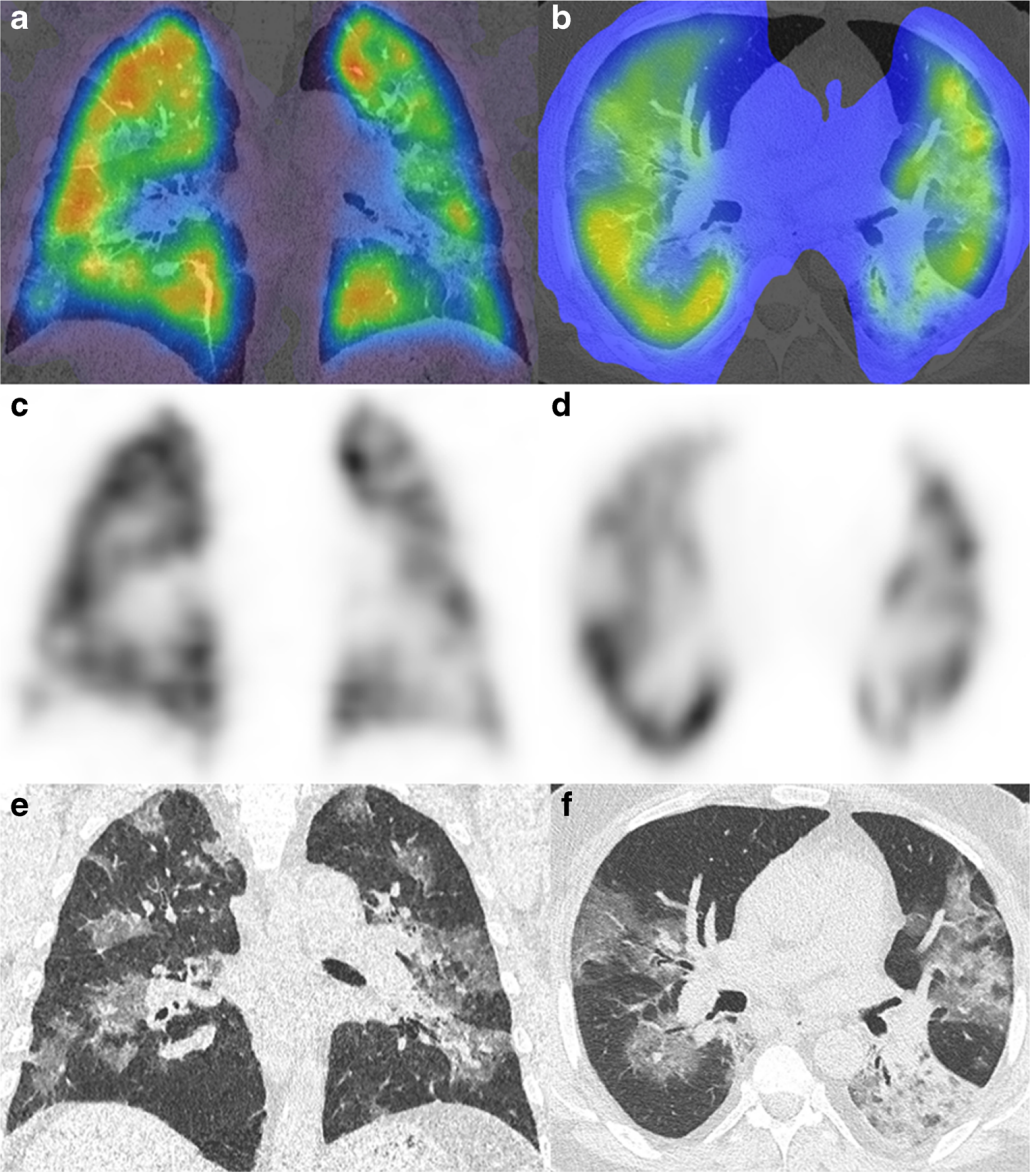
Perfusion SPECT/CT images of a heterogenous perfusion pattern in a case of COVID-19 pneumonia. Shown are the hybrid images (**a**/**b**), the perfusion SPECT (**c**/**d**), and the non-contrast enhanced CT images (**e**/**f**)

In recent case reports, V/Q-SPECT/CT has emerged as an alternative method to CT-angiography to exclude pulmonary embolism in cases where CT-angiography could not be performed [107, 112, 113]. Some authors present various examples of case findings in V/Q-SPECT/CT, reporting about the great usefulness of lung scintigraphy to detect pulmonary embolus in patients with COVID-19. Others report on instances of patients with COVID-19 pneumonia in whom the ventilation SPECT showed a heterogeneous pattern with preserved perfusion of the lung parenchyma, even in the presence of ground glass infiltrates to combined CT [112, 114]. In other cases, patients presented heterogenous perfusion patterns, where perfusion defects correlated with CT infiltrates, as shown in Fig. 6 [112, 115]. Nevertheless, co-registered native CT was helpful in cases of perfusion defects to exclude pulmonary embolism and to match perfusion heterogeneities of the scintigraphy to pulmonary infiltrates, which are frequent findings in lung CT-scans of patients with COVID-19 pneumonia [107, 108, 116]. Thus, the workup of patients with COVID-19 pneumonia benefits from having CT co-registered to the V/Q-SPECT and seems especially useful if only the perfusion SPECT is performed, rather than perfusion and ventilation SPECT.

### Cardiovascular imaging findings

#### Cardiovascular imaging in the initial phase of illness

In the initial phase of a SARS-Cov2-infection, the severity and prognosis of the disease depend on the degree of pulmonary involvement and on the presence or absence of acute myocardial damage and multiorgan dysfunction [117, 118]. Older patients [119], patients with diabetes [120, 121], and those with arterial hypertension [122] have a higher risk for a severe course of COVID-19. Additional risk factors include preexisting cerebrovascular and cardiovascular disease [123, 124], irrespective of gender, arterial hypertension, or diabetes [125]. Diagnostic procedures in the initial phase of the disease need to focus on physical examination, anamnesis, and the analysis of clinical history and laboratory biomarkers, whereas imaging mainly becomes necessary for determining the presence and severity of pulmonary and cardiac involvement by chest radiography, pulmonary CT [19], and echocardiography [126]. Other cardiac imaging modalities, such as coronary-CT, nuclear imaging, and cardiac magnetic resonance imaging, are more frequently used in the subacute, convalescent, and chronic phases of SARS-CoV2-infection [127].

Acute myocardial damage is present in 11–16% of COVID-19 patients with a severe course of disease, as indicated by elevated cardiac high-sensitivity troponin T (hs-TnT) levels in plasma [128, 129]. Certainly, disease-related myocardial damage is associated with a reduced long-term survival [124, 130] in patients with and without pre-existing coronary artery disease (CAD) [131]. The assessment of acute myocardial damage is based on the presence in plasma of elevated cardiac biomarkers (i.e., troponin, creatine kinase), abnormal heart failure biomarkers (i.e., B-type natriuretic peptides (BNP), NT-proBNP), and abnormal 12-lead electrocardiogram (ECG) suggestive of ischemia or myocarditis. In case of suspected acute coronary syndrome, patients should undergo immediate invasive coronary angiography [127]. If the patient shows signs of left and/or right ventricular dysfunction, hemodynamic instability, or suspected pericardial effusion, transthoracic echocardiography is the first-line imaging modality [126].

For safety reasons, most centers have segregated their imaging wards into separate sections for COVID-positive and non-COVID-19 patients [13, 132]. Transthoracic echocardiography procedures should be kept as brief as possible, to minimize the exposure of healthcare personal to the pathogen. This procedure includes a focused assessment of right and left ventricular global systolic function and regionalities, as well as analysis of valvular dysfunction and pericardial disease. Transesophageal echocardiography puts the performing physician at risk of contamination due to the aerosol generation that often occurs during intubation of the probe [133], and the necessity of this procedure should thus be carefully considered [134]. Cardiac CT, including an arterial and venous phase scan, may present a safer alternative in SARS-CoV2 patients to exclude non-invasively left atrial appendage thrombus due to atrial fibrillation before making a decision to undertake converting or ablation procedures [135].

#### Cardiovascular imaging in the subacute and convalescent phase

The pathologic mechanisms by which SARS-CoV-2 can cause myocardial injury remain elusive. Possible causal factors include myocardial injury resulting from direct damage to the cardiomyocytes by the virus, systemic inflammatory responses, immune-mediated response by interferon and cytokines, inflammation of the endothelium (i.e., endotheliitis) with subsequent endothelial dysfunction [136], and direct coronary plaque destabilization or microthromboembolism, with consequently reduced myocardial perfusion [137].

Endothelial dysfunction in COVID-19 occurs following the entry of SARS-CoV-2 particles into endothelial cells via docking to the ACE2 transporter/receptor [137]. This process induces endothelial inflammatory processes that provoke plaque instability, as well promoting a pro-thrombogenic state [138], which can lead to acute coronary syndrome, or to systemic and pulmonary embolisms [139]. ACE2, now also known to be the SARS-CoV-2 functional receptor, are found not only in the epithelia of the lung and small intestine but also in arterial and venous endothelial cells and arterial smooth muscle cells in all organs studied, including the brain [140]. In a Dutch university hospital, 31% of intensive care unit (ICU) patients with proven COVID-19 pneumonia had arterial or venous thromboembolic complications [141]. Increased plasma D-Dimer levels and prothrombin time were associated with greater disease severity and independently predicted a poor prognosis [142], with an increased risk for fatal outcome [143].

Pulmonary embolism occurred in 22–30% of patients with severe clinical features and oxygenation levels <92% who underwent contrast enhanced chest CT [106, 144, 145]. In a French multicenter study, pulmonary CT angiography was performed in 1240 of 2878 consecutive patients (43%) hospitalized for SARS-CoV-2 infection, among whom pulmonary embolism was found in 103 cases (8.3% of those with angiography and 3.6% of all COVID-19 patients) [146]. Beside venous thromboembolism, some authors hypothesized that direct pulmonary artery thrombosis may arise due to the severe lung inflammation and the hypercoagulable state in patients [147]. Pulmonary CT angiography is the best imaging modality to exclude non-invasively pulmonary embolism [148], irrespective to the exact pathomechanism of venous thromboembolism or direct pulmonary artery thrombosis.

**Fig. 7:**
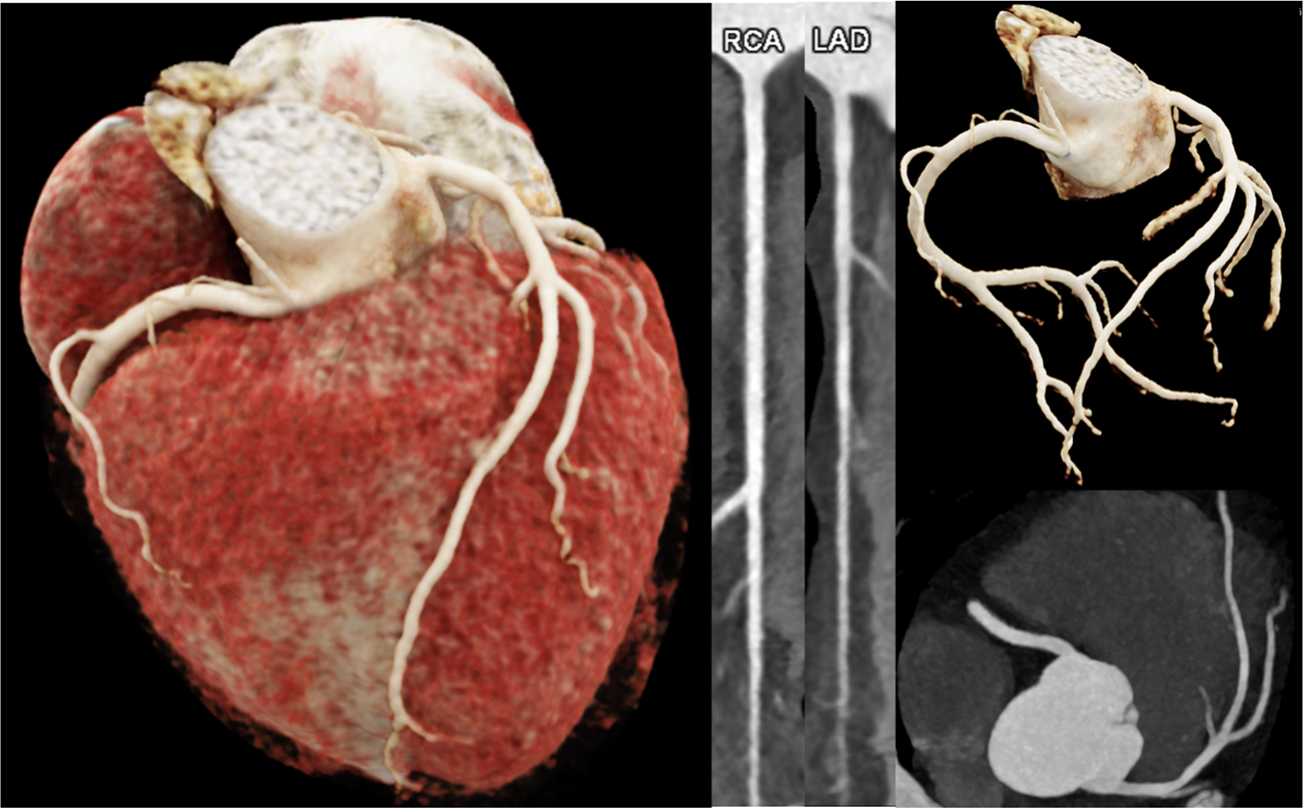
The heart of a 64-year-old female patient who had survived a SARS-CoV-2 infection 4 months previously. Now suffering from dyspnea and chest pain. cvRF, dyslipidemia. Results: CaSc 0, no stenosis

Thromboembolic complications of COVID-19 in the systemic arterial circulation system include thromboembolism to the upper and lower extremities [149], bowel ischemia [150], and stroke or myocardial infarction [151]. While invasive coronary angiography is routinely performed in patients presenting with an acute coronary syndrome, coronary CT angiography (CTA) presents an alternative non-invasive imaging modality to rule out significant coronary artery stenosis in patients with a low to intermediate pretest probability for coronary artery disease [152] (Fig. 7).

Coronary CTA may be performed with retrospective ECG-triggering or prospective ECT-gated one-step acquisition, if a CT scanner with high-pitch mode or large detector coverage is available [153, 154]. Dilatation of the coronary arteries with nitroglycerine spray and heart rate control with a beta-blocker improves the diagnostic accuracy of the procedure [155, 156]. CT coronary angiography may be combined with a thoracic CT angiography as a one-stop shop to rule out pulmonary parenchymal involvement, pulmonary embolism, and coronary artery disease [157, 158].

In patients with elevated cardiac troponin levels and no coronary artery disease, myocardial damage may be mediated by factors such as systemic inflammatory processes, cytokine storm, multi-organ-dysfunction, or direct myocardial infiltration of the virus [159, 160]. There are case reports of SARS-CoV-2 patients presenting with myocarditis [161–164], myocardial infarction with non-obstructive coronary arteries (MINOCA), or stress-induced Takotsubo cardiomyopathy [165]. In a recent publication on 388 MINOCA patients (not related to SARS-CoV-2), cardiac MRI supported a final diagnosis in 74% of cases. The final diagnosis was assigned in equal proportions (25%) to myocarditis, myocardial infarction, cardiomyopathy, and a normal cardiac MRI. Patients with cardiomyopathy (of whom 43% had Takotsubo cardiomyopathy) had a higher mortality than those with any other diagnosis, especially when the cardiomyopathy was combined with ST-elevation on ECG [166]. Whether such associations shall hold true as well for SARS-CoV2 patients warrants investigation. According to a position statement by the Society of Cardiovascular Magnetic Resonance (SCMR), cardiac MRI in COVID-19 patients should be focused on ventricular morphology and function, as well as myocardial tissue characterization [167, 168].

#### Cardiovascular imaging in the chronic phase

**Fig. 8:**
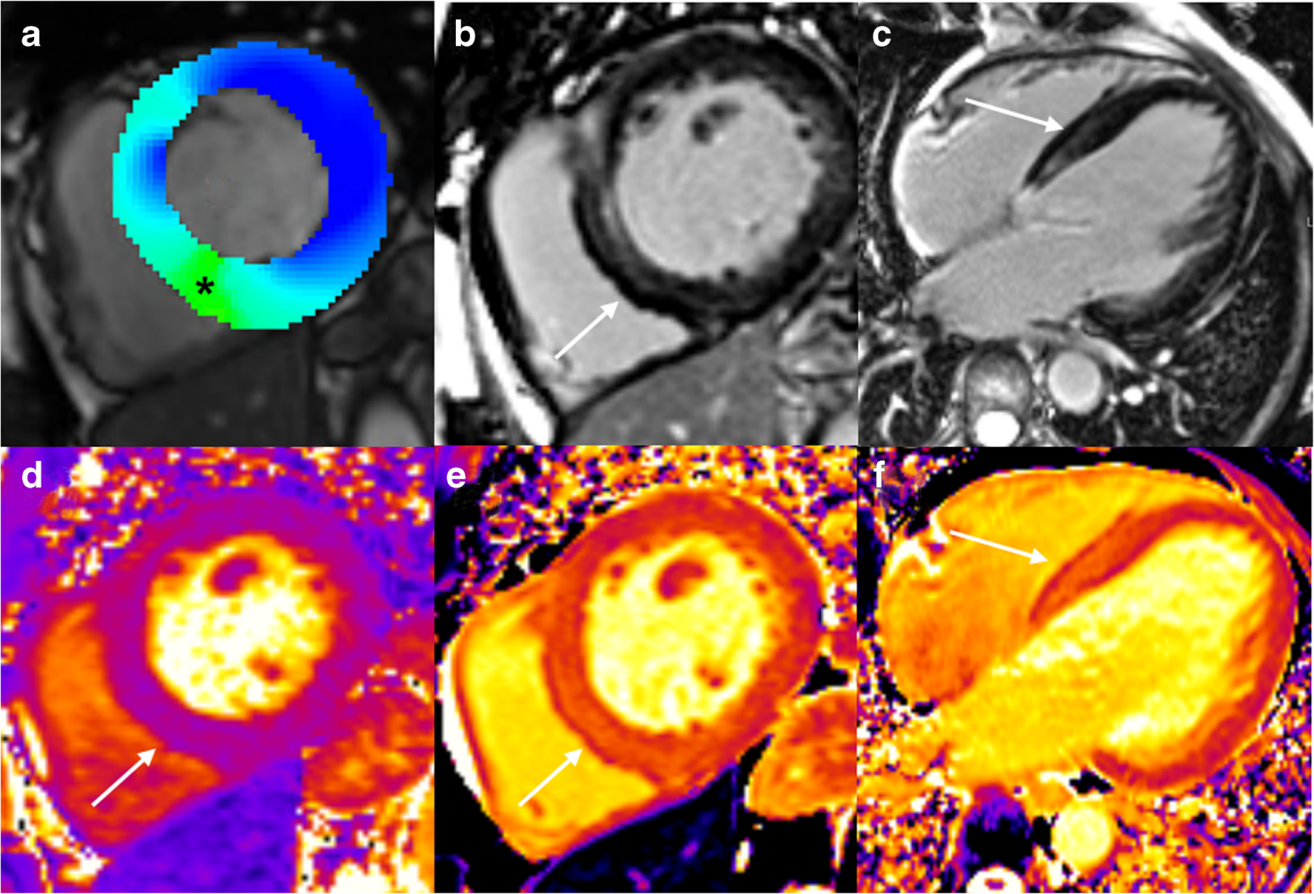
A 49-year-old male patient. New onset of dyspnea NYHA III since a SARS-CoV-2 infection 5 months ago. Cardiac MRI at 3T showing a dilated, eccentrically hypertrophied left ventricle (LV-EDV 151 ml/m^2^, LV mass 127 g/m^2^). Left ventricular (LV) ejection fraction (LV-EF 37%), LV peak circumferential (−12%), radial (18%), and longitudinal strain (−14%) were globally reduced, while the lowest regional peak circumferential strain was found in the LV septum (−6%, asterisk, **a**). Mid-ventricular Late-Gadolinium-Enhancement (LGE, **b**, **c**) was present in the LV septum, with normal T2 relaxation time (40 ms, **d**) in T2 mapping, but prolonged T1 relaxation time (1330 ms, **e**, **f**), consistent with myocardial fibrosis (white arrows)

We know little about the chronic phase of SARS-CoV2 infection and the degree to which late cardiovascular consequences are to be expected. In patients who recovered from a SARS-CoV-2-infection with pulmonary involvement, right ventricular dysfunction and possible secondary tricuspid regurgitation sometimes occurs due to pulmonary fibrosis with subsequent pulmonary-artery hypertension [169]. In addition to impaired right-ventricular function, some patients who had recovered from SARS-CoV-2 showed ongoing myocardial inflammation, diffuse myocardial fibrosis, and Late-Gadolinium Enhancement (LGE) [169] (Fig. 8). In one study with 100 patients who had recovered from a recent SARS-CoV-2 infection, cardiac MRI revealed cardiac involvement in 78 patients (78%) and ongoing myocardial inflammation in 60 patients (60%), examined 2–3 months after the positive SARS-CoV-2 test [170]. Those patients showed elevated T1 and T2 mapping values, both ischemic and non-ischemic patterns of myocardial LGE, as well as reduced left and right ventricular ejection fractions [170].

The link between myocardial inflammation and diffuse myocardial fibrosis is well-known [171]. Diffuse myocardial fibrosis may lead to left ventricular dysfunction and heart failure [172]. Ongoing myocardial injury, pericardial inflammation, microvascular ischemia, and fibrosis related to SARS-CoV-2 infection are associated with ventricular tachycardia [160, 173] and atrial fibrillation (AF) [174]. In a similar manner, inflammatory processes provoked by SARS-CoV2 may induce long-term vascular damage and accelerate the formation of atherosclerotic plaques in the coronary arteries and the aorta, which is a theme yet to be investigated in long-term follow-up studies [175].

#### Molecular cardiovascular imaging

Nuclear medical imaging methods such as myocardial perfusion imaging/MPI do not yet play a relevant diagnostic role in the context of COVID-19, as dictated by logistical and social factors. According to a joint statement of the American Society of Nuclear Cardiology (ASNC) and the Society of Nuclear Medicine and Molecular Imaging (SNMMI) from March 2020, all non-urgent diagnostic imaging studies, including nuclear cardiology procedures, should be postponed in order to minimize the spread of COVID-19 and to conserve hospital resources in the face of the pandemic [176, 177].

The effect of implementing these measures was evident in an Italian study showing a significant reduction in the number of stress SPECT-MPI studies during the COVID-19 pandemic compared with the corresponding months of the previous 3 years, whereas there was no difference in the prevalence of abnormal SPECT-MPI studies between the study periods [178]. The authors concluded abnormal imaging tests are likely missing for many heart patients during the pandemic. Furthermore, an international survey extending from April 16 to May 3 2020, which included responses from 72 countries, reported a 66% decline in the number of myocardial studies [179]. This result was recapitulated in other studies showing decreasing case numbers [180–182].

To facilitate an assessment of the urgency and justification for undertaking SPECT-MPI studies, the ASNC and SNMMI have given guidance using a three-level system for prioritizing nuclear cardiac examinations for different indications [176]. Especially for patients with confirmed or suspected COVID-19, referring physicians are advised to discuss the matter with a nuclear cardiologist or physician and to rationalize the absolute urgency for ordering an MPI or other procedures [183]. If other simpler imaging modalities are available for resolving clinical questions, they should be used preferentially. Thus, nuclear cardiology imaging in COVID-19 patients should be restricted to indications with no adequate alternative imaging modality, and having direct clinical consequence, for instance the urgent use of FDG-PET/CT for suspected infective endocarditis in the setting of prosthetic valves or intracardiac device infection [158, 176].

If it proves necessary to conduct an FDG-PET/CT examination, the ASNC/SNMMI guidelines include special recommendations for measures at nuclear cardiology facilities, in addition to the standard procedures like masking, social distancing, and hand hygiene [176]. These included selecting a protocol with the shortest possible scanning duration and least patient exposure time to staff, as well as the preferred use of first/stress only in single-day imaging protocols [176, 177]. Furthermore, manual blood pressure measurements should be avoided [133, 176, 177].

As the SARS-CoV-2 virus spreads via aerosol droplets, procedures that might involve patient coughing or otherwise releasing aerosols are high risk [183]. Therefore, exercise stress testing for MPI should be avoided and pharmacological stress, e.g., with vasodilators, should be preferred [176, 183, 184]. Since the A2A adenosine receptor agonist vasodilator Regadenoson only requires a 10-s infusion calling for close proximity of staff to the patient, it is the preferred stress agent, if not medically contraindicated [176]. Otherwise, it could be possible to use extra-long tubing to maximize the distance between staff and patient when administering adenosine or dipyridamole [176]. If exercise stress testing is deemed necessary in settings with moderate to high prevalence of active COVID-19, virus testing of the patient is recommended prior to the examination, and exercise protocols should be kept as brief as possible [133]. Moreover, if [^82^Rb] or [^13^N]-ammonia myocardial perfusion PET is available, it is preferable to myocardial perfusion scintigraphy due to its better efficiency, such that the complete rest-stress study acquisition last only 30 to 45 min [133, 177].

However, there are also circumstances and indications when nuclear medical procedures entail a lower risk of SARS-CoV-2 transmission compared to other diagnostic methods. For example, in febrile patients with bacteremia and suspicion of endocarditis, clinicians should consider FDG-PET/CT as a safer alternative to transesophageal echocardiography, which is apt to provoke considerable aerosol and droplet release [176].

Overall, pathologies associated with COVID-19 or suspicious for COVID-19 have generally been incidental findings on cardiological nuclear imaging, especially in SPECT/CT-MPI. In one case report, a man with a history of hypertension and dyspnea on exertion was referred for preoperative risk stratification prior to renal surgery [185]. After exercise stress testing, MPI with [^99m^Tc]-Sestamibi showed stress-induced ischemia in the apical septal segment. Furthermore, there were multifocal ground glass opacities with pathologically increased uptake of [^99m^Tc]-Sestamibi in both lungs, which were interpreted as typical features of COVID-19 lung involvement. However, there was no confirmation of the suspected infection by testing for SARS-CoV-2. Nevertheless, the patient was placed under quarantine and monitored. In another case report, myocardial perfusion scintigraphy with ^99m^Tc-Sestamibi was performed on a D-SPECT® camera, which showed normal gated stress images but also high signal in both lungs [186]. An additional CT in the patient showed ground-glass opacity and a crazy-paving pattern suggestive of COVID-19, with subsequent confirmation by RT-PCR analysis of a nasal swab.

In a study from the UK, 160 [^18^F]-FDG-PET/CT scans in asymptomatic patients and those with symptoms not primarily suggestive of COVID-19, but dating from the period of the lockdown, were reviewed retrospectively for incidental findings in the lungs and in extrapulmonary sites [187]. Among these 160 cases, one showed focal right ventricular uptake, one a patchy right ventricular uptake, and another had one diffuse right ventricular uptake, suggesting perhaps 2% incidental positivity. However, there was no systematic testing for SARS-CoV-2 for these patients. In another study, the aim was to assess by [^18^F]-FDG-PET/CT the inflammatory status at the presumed peak of the inflammatory phase in non-critically ill patients (*n* = 13) admitted for COVID-19 [188]. Patients enrolled prospectively underwent an [^18^F]-FDG PET/CT examination from day 6 to day 14 after onset of symptoms. Only one of these patients had significant physiological myocardial FDG uptake, even though there had been no intervention to suppress physiologic myocardial glucose metabolism prior to scanning, such that one might normally have expected substantial myocardial tracer uptake in most individuals. The authors suggested that the myocardial metabolic pathway may disfavor glycolysis during COVID-19 infection, perhaps due to a loss of sympathetic tone that would otherwise promote myocardial FDG uptake [189], a conjecture that might be tested by examination of cardiac variability or perhaps by [^123^I]-MIBG scintigraphy.

In another case report, a 57-year-old COVID-19-positive man with no history of cardiovascular disease had ground-glass opacities on CT detected on the day of hospital admission [190]. Due to his poor response to symptomatic treatment, worsening symptoms of chest tightness, and palpitations, the patient underwent [^18^F]-FDG-PET/CT and [^18^F]-FDG-PET/MRI examinations. The [^18^F]-FDG-PET/CT showed focal ground-glass opacities in both lungs without hypermetabolism, suggestive of the absorption phase of SARS-CoV-2 infection, and revealed normal-sized but hypermetabolic and probably inflammatory mediastinal lymph nodes. A subsequent [^18^F]-FDG-PET/MRI did not show any pathological cardiac findings, but did reveal a diffuse increase in hepatic [^18^F]-FDG-uptake consistent with a systemic inflammatory response. Due to the lack of cardiovascular involvement, the authors suggested that their case study did not indicate a clear association between the tissue distribution of ACE2 and organ damage [190]. As ACE2, which enables the entry of SARS-CoV-2 into cells, also has high expression in the heart, cardiac tissue is likely to be vulnerable to the infection. In a case report of a male cardiology patient from a north Italian COVID-19 high-risk region, [^18^F]-FDG-PET/CT was used to check for superficial and deep lead tract infection of his left ventricular assist device. Results showed an inflammatory pattern at the second and third level (superficial tract) of driveline tunneling suggestive of a locally active infection and potential evolution into a subcutaneous abdominal fistula [191]. Furthermore, there were incidental findings of multilobular subpleural ground-glass opacities in the lung with increased [^18^F]-FDG-uptake that were suggestive of COVID-19. A subsequent RT-PCR test was negative, but a second test 8 days later was positive for SARS-CoV-2.

In conclusion, nuclear cardiology imaging is not presently playing a major role in the evaluation of cardiac involvement in COVID-19. Nevertheless, practitioners should be alert to the possibility of incidental pulmonary findings, especially since patients with cardiologic illnesses are a high-risk group in the event of COVID-19 infections. Furthermore, guidelines call for adaptation of imaging procedures to minimize the risk of transmission between patients and medical staff.

### Neurological imaging findings

#### Conventional imaging

Central nervous system (CNS) involvement is a known complication of viruses with neurotropic characteristics. There are also reports of neurological symptoms in patients with SARS-CoV-2 infection, indicative of the neurotropic nature of the virus [192]. Adverse neurological symptoms have occurred in the form of anosmia, hyposmia, dysgeusia, headache, nausea, vomiting, agitation, delirium, and impaired consciousness [192–196]. Coronaviruses (CoVs) are generally neurotropic [197], and both SARS-CoV and MERS-CoV have occasionally caused clinically relevant CNS infections [198–202], while SARS-CoV particles have been detected in neurons of the human brain [203]. It is also likely that SARS-CoV-2 may gain access to the CNS where it can induce neuronal injury. Three hypotheses of SARS-CoV-2 entry into the CNS have been discussed: (a) intranasal inoculation with spread via olfactory nerves and olfactory bulb to the brainstem; (b) transsynaptic spread from neuron to neuron via endocytosis/exocytosis, and (c) hematogenous spread via infected monocytes and passing the blood-brain barrier [204]. Pathophysiology of CNS manifestations from COVID-19 includes three mechanisms: (a) direct viral entry into the brain, (b) adverse immune response, and (c) respiratory stress [205].

**Fig. 9:**
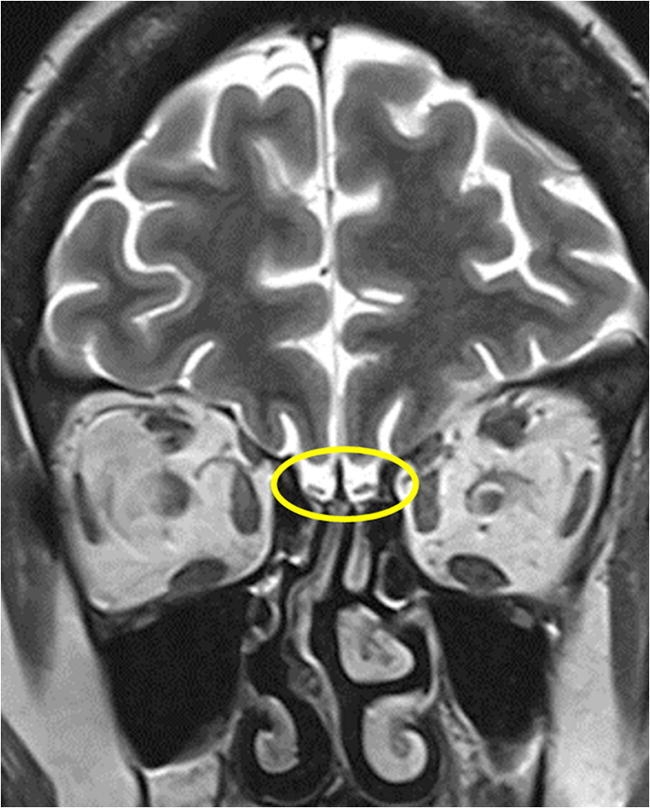
Coronal T2-WI MR image in a patient after COVID-19 and hyposmia demonstrates bilateral atrophy of the olfactory bulb (yellow circle)

Recent experimental evidence demonstrates that a human CoV strain, HCoV OC43, can travel from the nasal cavity to the olfactory bulb, and then spread to the piriform cortex and ultimately to the brainstem, via both passive diffusion and axonal transport [206, 207]. Animal studies have shown that the viral infiltration started in the olfactory bulb and progressively invaded subcortical and cortical regions. Anosmia was reported in 5.1% and ageusia in 5.6% of 214 COVID-19 cases in the first study from Wuhan [208]. A large multicenter European study reported olfactory impairment in 85.6% and gustatory dysfunction in 88% of 417 COVID-19 patients [209]. However, a recent study from Spain documented anosmia in only 4.9% and ageusia in 6.2% of 841 COVID-19 patients [210]. In an early post-mortem MRI study in COVID-19 non-survivors, olfactory bulb asymmetry and atrophy was detected in 4 of 19 (21%) subjects [211]. MRI signal alterations of the olfactory bulb (Fig. 9) along with bilateral atrophy and signal increase in the gyrus rectus were reported in a case study with examination at 4 days after the onset of anosmia [212].

In the first small series from the USA, five cases of large-vessel stroke in patients with severe acute respiratory syndrome were reported [213]. A retrospective study from Wuhan/China showed that the incidence of stroke among hospitalized patients with Covid-19 was approximately 2.8% [214]. The percentage of reported COVID-19-related strokes varies considerably in different patient series. In a French series of 58 patients, stroke was documented in 5% (3 of 58) [195], but only 2% (50 of 235) of patients had stroke in a Turkish study [215]. In a larger Spanish study, 50 of 841 of COVID-19 cases (1.3%) had cerebral large-vessel occlusions [210]. The highest incidence of COVID-19-related strokes was reported in an Italian study (34 of 119, 28.6%) [216]. In a retrospective cohort study of 3218 hospitalized COVID-19 patients in New York, 454 (14.1%) underwent neuroimaging, with stroke being the most common finding (92.5%) [217]. Large infarcts were detected in 44.5% of the cases, whereas lacunar infarcts were diagnosed in 24% [217]. It seems that strokes in SARS-Cov-2 infection may be the result of two different mechanisms, namely transient hypercoagulability and/or systemic vasculitis/endotheliitis [192, 204]. In recently published study from the UK, in 6 of 8 patients with stroke, D-dimer was significantly raised indicating hypercoagulability; four of the stroke patients had thrombus in intra- or extracranial vessels, and four had a pulmonary embolism [192].

Another scantly explored neurological manifestation of COVID-19 is meningitis/encephalitis. The first report on SARS-CoV-2-associated meningitis/encephalitis from Japan reported high signal intensity in the mesial temporal lobe [218]. Interestingly, RT-PCR was negative in the nasopharyngeal swab of that patient but was positive in the cerebrospinal fluid (CSF). A large French observational retrospective study reported unilateral FLAIR and/or diffusion abnormalities in the MRI in 43% of cases [195].

Once entering the CNS, the virus can induce a dysregulated host immune response known as “cytokine storm” [219]. The cytokine storm and the direct cytopathic damage by the virus particles may lead to neurological diseases, such as encephalitis, acute flaccid paralysis, or acute necrotizing encephalopathy (ANE) in susceptible individuals. In one case report from the USA, MRI showed hemorrhagic rim-enhancing lesions within the bilateral thalami, medial temporal lobes, and subinsular regions consistent with ANE [220], which is a well-known complication of influenza and other viral agents [221–223].

There are reports of acute disseminated encephalomyelitis (ADEM), cytotoxic lesions of the corpus callosum (CLOCC), transient encephalopathy, and leptomeningeal enhancement in association with COVID-19 [192, 195, 215, 224]. Clinically transient encephalopathy manifested as delirium and psychosis [192]. The negative RT-PCR in the CSF of such cases suggests an underlying mechanism from secondary processes rather than direct viral infection of the CNS. Miller-Fisher syndrome and Guillain-Barré syndrome have been reported to precede COVID-19 infections, suggesting a para- or post-viral process. *Haemophilus influenzae*, *Campylobacter jejuni*, ZIKA virus, and cytomegalovirus are the most common pathogens involved. Bickerstaff’s encephalitis overlapping with Guillain-Barré syndrome, intensive care unit-acquired weakness, or other toxic or infectious neuropathies have been reported during or after Middle East respiratory syndrome (MERS-CoV) treatment. Two patients infected with SARS-CoV-2 presented with Miller-Fisher syndrome and polyneuritis cranialis [225]. Serum GD1b-IgG antibodies were positive in one of the reported patients, supporting the hypothesis of immune-mediated injury rather than direct viral neurotropism.

### Molecular neuroimaging

Although respiratory diseases are clinically prominent in the context of infection with SARS-CoV-2, there are increasing reports of neurological manifestations [226]. Despite neurological imaging usually being an MRI/CT domain in patients with SARS-CoV-2 infections [227], there are cases where nuclear medical procedures (especially [^18^F]-FDG-PET/CT) have been used. For example, brain MRI, CSF testing, and [^18^F]-FDG-PET/CT were performed in the case of a 72-year-old man with a positive oropharyngeal swab test for SARS-CoV-2 who presented with a subacute cerebellar syndrome and myoclonus following general infectious symptoms. Brain MRI and CSF testing did not show any pathological findings, whereas brain [^18^F]-FDG-PET/CT showed diffuse cortical hypometabolism along with hypermetabolism in the putamen and cerebellum, consistent with encephalitis, and especially cerebellitis. Furthermore, this patient had high serum and CSF titers of IgG autoantibodies against the nuclei of Purkinje cells as well as against striatal and hippocampal neurons. The case revealed a possible relationship between SARS-CoV-2 infection and autoimmune encephalitis, where [^18^F]-FDG-PET/CT had diagnostic advantages over brain MRI [228].

Another recent publication included four patients with encephalopathy related to COVID-19 (confirmed by a positive RT-PCR assay from a nasopharyngeal swab) [229]. The patients, who were aged at least 60 years, showed various degrees of cognitive impairment mainly suggestive of frontal lobe disorders, which were associated in part with anosmia (*n* = 2), cerebellar syndrome (n = 2), myoclonus (*n* = 1), psychiatric manifestations (*n* = 1), and status epilepticus (*n* = 1). None of the four patients showed signs of encephalitis on MRI and CSF analyses, including RT-PCR for SARS-CoV-2, did not show any significant abnormalities. On [^18^F]-FDG-PET/CT, all four patients showed a common pattern of hypometabolism in the prefrontal or orbito-frontal cortices and hypermetabolism in the cerebellar vermis, despite their distinctly different neurological presentations. The authors supposed that the central focal neurological signs or seizures, the absence of meningitis and of SARS-CoV-2 in the CSF, and the [^18^F]-FDG-PET/CT findings were suggestive of a parainfectious cytokine-storm with post-infectious autoantibody- or cell-mediated immune mechanisms rather than a direct viral neuroinvasion. They further argued that the elevated CSF level of interleukin-6 (in both patients examined for this), as well as the clinical improvement after immunotherapy, was indicative of a transient immune process [229]. Nevertheless, in the absence of a control group, the possibility of a spontaneous amelioration cannot be excluded.

Neurological complications can arise from other respiratory viruses, especially seasonal and pandemic influenza [206]. Two prominent examples are anosmia and ageusia, which are very common neurologic symptoms of COVID-19 appearing either in isolation or together with other features [226, 230, 231]. These sensory deficits are often an initial manifestation of the disease [232]. Although such symptoms can occur in any respiratory infection, simply due to rhinitis, their isolated occurrence prior to COVID-19 onset strongly suggests an involvement of the olfactory nerve [226]. Furthermore, these sensory symptoms were noted more frequently among COVID-19 patients than for a historical influenza patient cohort [232].

An [^18^F]-FDG-PET/CT examination was performed in a 27-year-old right-handed woman, who had been earlier diagnosed with COVID-19 by RT-PCR and suffered from isolated anosmia persisting for 6 weeks [233]. The scan showed pathological hypometabolism of the left orbitofrontal cortex [233, 234], a region which receives projections from the primary olfactory cortex [235, 236]. Hyposmia and anosmia due to CNS damage of other etiologies have previously been widely investigated by molecular imaging, especially in the context of Parkinson’s disease [237–239], where anosmia is an early presenting symptom.

**Fig. 10:**
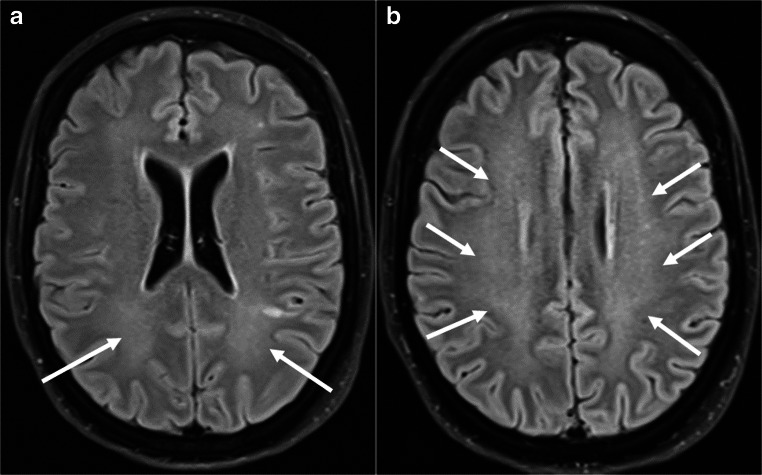
**a**, **b** A 44-year-old female patient with COVID-19 and severe ARDS. Axial FLAIR MR images show bilateral, ill-defined hyperintensity of the white matter consistent with leukoencephalopathy (white arrows)

Iatrogenic neurological complications may occur in the context of therapy for pre-existing conditions. In a recent case, leukoencephalopathy emerged after long-term administration of Tocilizumab in a COVID-19 patient with rheumatoid arthritis. The patient showed leukencephalopathy on brain MRI, with a lactic acid peak on magnetic resonance spectroscopy in the left temporal lobe, decreased cerebral perfusion in SPECT, and hypometabolism of the left frontal lobe in [^18^F]-FDG-PET/CT [240]. Figure 10 shows an example of leukoencephalopathy.

Tocilizumab is a monoclonal antibody directed against the interleukin-6 receptor, which has also proposed as a treatment to mitigate the cytokine-storm syndrome sometimes associated with severe COVID-19 [241]. Indeed, Tocilizumab treatment has apparently imparted reduced mortality of patients with COVID-19 requiring intensive care unit support [241]. Nonetheless, the potential for adverse neurological effects of Tocilizumab should be considered when treating COVID-19 patients.

Although molecular imaging currently plays a minor role in neuroimaging of SARS-CoV2 infections, it might yet gain importance in the diagnosis of autoimmune encephalitis once the clinical use of [^18^F]-FDG-PET/CT for this indication becomes more widely appreciated [239, 242]. This procedure could prove very helpful in the diagnostic workup, as there are known cases of likely autoimmune (steroid responsive) but seronegative encephalitis and conversely of seropositive autoimmune encephalitis in the context of SARS-CoV-2 infection, with absence of any alterations on MRI [228, 243]. As far as may be judged from the few available case reports, hypometabolism on [^18^F]-FDG-PET/CT could serve as a cerebral quantitative biomarker of neuronal involvement [244, 245] and should therefore be considered especially in COVID-19 patients presenting with anosmia or any acute central nervous system impairment [229]. However, larger viral and post-viral cohort studies shall be required to confirm any relationship between pathological findings on [^18^F]-FDG-PET/CT and distinct neurological disorders such as persistent cognitive or emotional disturbances as well as pain syndromes. Furthermore, longitudinal [^18^F]-FDG-PET/CT studies could prove to be useful to determine whether pathological PET findings are attributable to a transient functional inactivation or rather to irreversible brain damage [229]. We furthermore note the precedent set by [^18^F]-FDG-PET findings in patients with chronic fatigue syndrome [246], which is arguably a post-viral condition. As such, [^18^F]-FDG-PET may assume a growing importance in the years to follow the present pandemic.

### Additional organ manifestations of COVID-19

Symptomatic patients suffering of COVID-19 typically present with symptoms caused by acute lung injury such as fever, cough, and dyspnea. However, rising testing capacities and case numbers have led to the emerging understanding of COVID-19 as a systemic disease, affecting not only the pulmonary, cardiovascular, or neurological systems, as reviewed above, but also extending to other systems such as the renal, gastrointestinal (GI), or the hepatobiliary system. Against this background, there is an increasing awareness of these manifestations and their associated imaging findings.

The pathophysiological mechanism of abdominal organ damage by COVID-19 derives from the type II pneumocytes of the lung. As in the case of pulmonary and vascular infections, the virus probably gains access to visceral organs via their surface expression of ACE2. This docking site has broad expression in the human viscera, thus enabling direct or indirect tissue damage of the liver, biliary ducts, pancreas, spleen, intestine, or kidneys [118, 247–253]. A hepatocellular injury pattern is assuming an increasing clinical importance, now evident in 14–53% of critically ill COVID-19 patients [122, 254–256]. It shall be important to follow-up patients receiving investigational antiviral treatments such as remdesivir, lopinavir, or tocilizumab, due to their potential for causing drug-induced liver injuries [257]. The ultrasound findings of hepatobiliary manifestations of COVID-19 reported in the literature include signs of biliary stasis such as gallbladder sludge and distension, as well as ascites [258–260]. Occasionally, CT findings of acute cholecystitis or pancreatitis have been reported [260]. However, it seems very likely that CT, MRI, or ultrasound examinations may prove to detect features of hepatic steatosis in COVID-19 patients.

Gastrointestinal manifestations in COVID-19 patients are also of great clinical importance, considering that bowel wall abnormalities were a frequent finding upon admission to an ICU [258]. The incidence of gastrointestinal involvement ranges from 12 to 61% in the literature. Although being more frequent among patients with longer duration of COVID-19 illness and ICU submission, gastrointestinal findings have not yet been associated with increased mortality [248, 255, 258, 261–263]. Some studies describe a small percentage of patients in whom the gastrointestinal symptoms preceded the pulmonary manifestations, or who presented only with symptoms such as nausea, vomiting, diarrhea, or abdominal pain [167, 225, 248, 263].

The most commonly reported gastrointestinal imaging finding is bowel wall thickening, which can include all parts of the bowel [258–260]. In the COVID-19 cohort of Bhayana et al., bowel wall thickening was associated with admission to the ICU, and small bowel thickening was an exclusive finding in ICU patients [258]. Others have reported various imaging findings, including intestinal pneumatosis, pneumoperitoneum, portal vein thrombosis, and portal venous gas, all of which are associated with mesenteric ischemia [258–260]. A possible explanation for these findings may be the high expression of ACE2 not only in enterocytes but also on the vascular endothelium, making the visceral structures highly susceptible towards infection by the virus. The occurrence of microvascular small-bowel injury is suggested by the histopathological presence of diffuse endothelial inflammation in the submucosal vessels of the small intestine in COVID-19 patients [264]. Furthermore, the infection can give rise to a systemic coagulopathy, especially in critically ill patients [265], as described in the preceding sections. Occasional gastrointestinal findings include ascites, fluid-filled colon, colonic ileus, and cases of ileocolic intussusception [102, 266–268]. In consideration of these possibly life-threatening complications and of the fact that COVID-19 patients with primary gastrointestinal symptoms often experience delayed diagnosis, there is a need for vigilance about the need for imaging in patients with these manifestations [248, 269].

Acute kidney injury (AKI) presenting with proteinuria and hematuria is a severe possible complication of COVID-19, which imparts high mortality [270–273]. In a study from the USA, there was a 37% incidence of 37% of AKI among COVID-19 patients, of whom one-third had diagnosis within 24 h after admission, and 14% even required dialysis [272]. Renal pathophysiology is probably multifactorial, but SARS-CoV-2 may directly target renal cells via ACE2, as is supported by certain histopathological findings [118, 253]. Another possibility is that AKI is caused by cytokine-storm during the infection, as can occur during infections with influenza and other viruses [274]. Of course, other potential etiologies of AKI in critical illness situations also remain relevant, among which are organ volume depletion, interstitial nephritis, ARDS, or rhabdomyolysis [275]. Renal imaging findings are rarely reported in the COVID-19 literature, the most common being signs on MR of renal infarction, namely sharply demarcated, possibly patchy hypoenhancing or non-enhancing areas in the kidney [276–278]. In pathophysiological terms, this kind of lesion is most probably attributable to the global coagulopathy that can occur in COVID-19 [265, 279].

Although myalgia is a commonly reported symptom among COVID-19 patients [280, 281], musculoskeletal manifestations are most probably underdiagnosed, such that so far only one imaging result is yet available in the literature, being an MR study of acute myositis [282]. We emphasize that new COVID-19 manifestations such as peripheral nerve disease or neuromusculoskeletal disorders seem likely to emerge with time, in keeping with the precedent set by SARS-CoV-1. However, there are not yet reports of such findings in SARS-CoV-2 patients [283, 284].

Another rare manifestation of COVID-19 is cutaneous lesions including maculopapular rash, vesicular lesions, and livedoid/necrotic lesions, with the latter possibly being associated with a more severe course of COVID-19 [285]. A single-center observational study from Italy first reported that dermatologic manifestations occurred in 20% of patients hospitalized with COVID-19 [286]. There was no significant correlation between severity of respiratory symptoms and dermatologic manifestations, and there are not yet any specific imaging findings of cutaneous manifestations reported in the literature.

### Artificial intelligence in the diagnosis of COVID

Recent advances in artificial intelligence (AI) and specifically in deep learning (DL), along with the availability of large datasets and powerful computational hardware based on graphics processing units (GPUs), have led to substantial changes in medical imaging. AI extends human powers of the perception of information in data, and may surpass human performance in some situations [287, 288]. AI based on the analysis of thoracic images has already contributed to the fight against the COVID-19 pandemic, particularly in assisting the diagnosis, stratification, prognosis, and treatment of COVID-19 patients [289–291]. AI-based systems for radiomic analysis of chest X-ray (CXR) images are playing a prominent role in the newly introduced approaches and are now finding application for lung ultrasound (LUS) images.

AI-based analysis of CRX images mainly focuses on the development of support systems for the rapid diagnosis and differentiation of COVID-19 from other types of pneumonia. Various DL-based approaches have been published [71, 292–294], but only a few of these approaches have been validated against radiologists. There was an investigation of the accuracy of a DL-based system on the two-class problem of discriminating COVID-19 vs other pneumonias on CRX images [295]. The algorithm in that study was pretrained on publicly available data and fine-tuned on data collected from four local medical centers. The performance of the trained system was then compared with consensus findings of three board-certified radiologists, which showed that the DL system had higher sensitivity but similar specificity as did the radiologists. A similar investigation using a larger dataset was presented in [296], where a DL-based system using an ensemble of networks was compared against three experienced thoracic radiologists. The results indicated that the system outperformed the experienced radiologists. The two-class diagnosis of COVID-19 vs no COVID-19 on CRX was addressed in [297], where an ensemble of convolutional neural networks was pretrained on publicly available data, fine-tuned on data collected from local clinical sites, and then compared with decisions by five experienced radiologists. The results showed that the DL-system was about as accurate as the consensus of the experienced thoracic radiologists. The three-class problem of COVID-19 pneumonia vs other pneumonia vs normal was addressed by [298], where the performance of a dedicated DL-system was compared against six readers. The system performance was comparable with that of six independent readers. In a more recent study [299], the performance of a newly introduced DL-based system trained on publicly available datasets was compared against 11 radiologists for the three-way discriminatory diagnosis of COVID-19 pneumonia vs. other pneumonias or normal. The system detected COVID-19 very accurately and outperformed radiologists at various training levels, while it was able to separate COVID-19 pneumonia from other types of pneumonia more accurately than were the human readers. Finally, in [300], a DL algorithm was introduced to calculate a measure of pulmonary disease severity on confirmed COVID-19 CXRs, with very encouraging results and in good agreement with follow-up CRXs.

Since the very beginning of the COVID-19 pandemic, several studies have examined the AI-based analysis of lung CT images. This was initially applied to the task of differentiating COVID-19 from other lung diseases [301, 302], and more recently to assess its severity [303] and prognosis [304, 305]. The initial attempts contained a limited number of cases without expert validation. Nevertheless, the results clearly indicated that AI technology is potentially valuable in the diagnosis of COVID-19 using CT imaging. In a recent study [306], the performance of a DL-based algorithm trained on publicly available CT images was compared against two experienced radiologists. The results indicated that the system was slightly superior to the radiologists. In [307], a CT-based triage system was introduced that was able to alert physicians when imaging features suggestive of COVID-19 pneumonia were detected. A DL system subsequently developed using a large data set performed remarkably well on an external validation set. Positive cases were identified by machine much more rapidly than by a radiologist, including the time require to draft and release the report. The affected lung areas identified by DL were also in good agreement with the radiologist’s report. Furthermore, the development and multicenter validation of the decision support system using clinical and laboratory variables along with radiological variables derived by CT images for severity risk assessment during hospitalization was presented in [308]. In that study, the results of the radiomic analysis were compared to the performance achieved by the pneumonia severity index (PSI), a clinical assessment method, indicating that the DL approach has the potential to be used for assessing the onset of severe and critical illness among COVID-19 patients.

LUS image analysis was proposed in [309] to evaluate the progression of COVID-19 pneumonia based on the severity of lung involvement. In [310], the authors trained a DL-based system on LUS collected at five clinical centers in Italy to predict the disease severity score proposed in [309]. In addition, others used a neural network to predict diagnostic outcomes [311]. The network was trained on publicly available videos and images and achieved promising results. None of these LUS systems has yet been validated against trained experts.

## Conclusion

Although there is a long history of nuclear imaging in infectious diseases of the chest, there is no current indication for the use of PET/CT in routine clinical diagnosis or management of COVID-19. However, in this still poorly understood disease, multi-modal imaging may shed light on the underlying pathophysiological processes involved. Furthermore, well-established nuclear medicine research tools such as the radiolabeling of immune or inflammatory cells for PET imaging [312, 313] serve as an example of how molecular imaging could assist in the development of innovative therapies.

We aimed in this comprehensive review article to provide an overview of the current knowledge on COVID-19 and imaging. It is apparent that SARS-CoV-2 infection is a systemic disease that can affect multiple organ systems besides the lungs. Radiological exams have played a pivotal role in the detection and diagnosis as well as patient management since the very beginning of the pandemic. The authors of this article gathered evidence from all across the field of medical imaging and summarized the state-of-knowledge on COVID-19 in late 2020 and early 2021. However, we acknowledge that knowledge is lacking for many aspects of COVID-19, including the imaging results for mid- and long-term effects, which is a research topic for the coming years.
